# Self-perceived vs actual and desired weight and body mass index in adult ambulatory general internal medicine patients: a cross sectional study

**DOI:** 10.1186/s40608-014-0026-0

**Published:** 2014-12-12

**Authors:** Kirsten G Mueller, Ryan T Hurt, Haitham S Abu-Lebdeh, Paul S Mueller

**Affiliations:** Division of General Internal Medicine, Mayo Clinic, 200 First St SW, Rochester, MN 55905 Minnesota; Division of Gastroenterology and Hepatology, Mayo Clinic, Rochester, Minnesota; Division of Endocrinology, Diabetes, Metabolism, and Nutrition, Mayo Clinic, Rochester, Minnesota

**Keywords:** Body mass index, Obesity, Overweight, Diagnosis, Patient education

## Abstract

**Background:**

No study has compared patients’ self-reported heights and weights (and resultant self-reported body mass indexes [BMIs]) with their actual heights, weights, and BMIs; their self-perceived BMI categories; and their desired weights and BMIs and determined rates of clinicians’ documented diagnoses of overweight and obesity in affected patients in a single patient group. The objectives of this study were to make these comparisons, determine patient factors associated with accurate self-perceived BMI categorization, and determine the frequency of clinicians’ documented diagnoses of overweight and obesity in affected patients.

**Results:**

A total of 508 consecutive adult general internal medicine outpatients (257 women, 251 men; mean age, 62.9 ± 14.9 years) seen at Mayo Clinic in Rochester, Minnesota, between November 9 and 20, 2009, completed a questionnaire in which they reported their heights, weights, self-perceived BMI categories (“underweight,” “about right,” “overweight,” or “obese”), and desired weights. These self-reported data were compared to actual heights, actual weights, and actual BMI categories (measured after the questionnaire was completed). Overall, 70% of the patients were overweight or obese. The average self-reported weight was significantly lower than the average actual weight (80.3 ± 20.1 kg vs 81.9 ± 21.1 kg; *P* < .001). The average self-reported BMI was significantly lower than the average actual BMI (27.6 ± 5.7 kg/m^2^ vs 28.3 ± 6.1 kg/m^2^; *P* < .001). Overall, 32% of patients had obesity; however, only 6% perceived they were obese. Accuracy of self-perceived BMI category decreased with higher actual BMI category (*P* < .001 for trend). Female sex, higher education level, smoking status, and lower BMI were associated with higher accuracy of self-perceived BMI category. Desired weight loss increased with higher self-perceived and actual BMI categories (*P* < .001 for trends). Of the 165 patients who actually were obese, only 40 (24%) had obesity documented as a diagnosis in their medical records by their clinicians. Statistical tests used were the paired t test, the Pearson χ2 test, the Cochrane-Armitage trend test, the Wald test of marginal homogeneity, analysis of variance, and univariate and multivariate logistic regression.

**Conclusions:**

Many obese patients inaccurately perceive their BMI categories; accuracy decreases with increasing BMI. Clinicians should inform patients of their BMIs and prescribe treatment plans for those with overweight and obesity.

## Background

More than one-third of US adults are obese [1]. Obesity has been associated with more than 60 diseases, including 12 different cancers [2]. If trends continue, obesity will overtake smoking as the leading preventable cause of death in the United States [3]. Given these data, the American Medical Association (AMA) now recognizes obesity as a disease that requires medical intervention [4].

Nonetheless, many people do not regard obesity as a disease [3,5] and have inaccurate perceptions of their own weight and body mass index (BMI) category (ie, underweight, normal weight, overweight, or obese) [6-26]. Lay perceptions of overweight and obesity have changed in recent decades to the extent that what was once considered “overweight” is now considered “about right” [10,16,17,19,27]. These observations are important because an overweight or obese person must recognize his or her unhealthy weight and its associated health risks before he or she will seek treatment [28].

Indeed, evidence suggests that obese women who accurately perceive their obesity (eg, “feel that their body size is too large”) are less likely to gain weight than obese women who inaccurately perceive their weight [14]. A patient’s report of being told by a clinician he or she has overweight or obesity is associated with realistic self-perception of weight, desire to lose weight, and attempts to lose weight [7]. A clinician’s diagnosis of obesity is strongly associated with a weight management plan [29,30]. Yet, many clinicians do not diagnose overweight and obesity in affected patients [29-33].

A number of studies have assessed self-perceived BMI category (ie, underweight, normal weight, overweight, and obese) in various populations [6-18,20-23]. However, few studies have compared self-perceived BMI category with actual BMI category [10-14,18,20,22], and only 2 have compared self-perceived BMI category with desired weight (and resultant desired BMI category) [8,21]. No study has compared patients’ self-reported heights and weights (and resultant self-reported BMIs) with their actual heights, weights, and BMIs; their self-perceived BMI categories; and their desired weights (and resultant desired BMIs) and determined rates of clinicians’ documented diagnoses of overweight and obesity in affected patients in a single patient group. The objectives of this study were to make these comparisons, determine patient factors associated with accurate patient self-perceived BMI categorization, and determine the frequency of clinicians’ documented diagnoses of overweight and obesity in affected adult ambulatory patients seen in a general internal medicine clinic at an academic medical center.

## Methods

**Figure 1:**
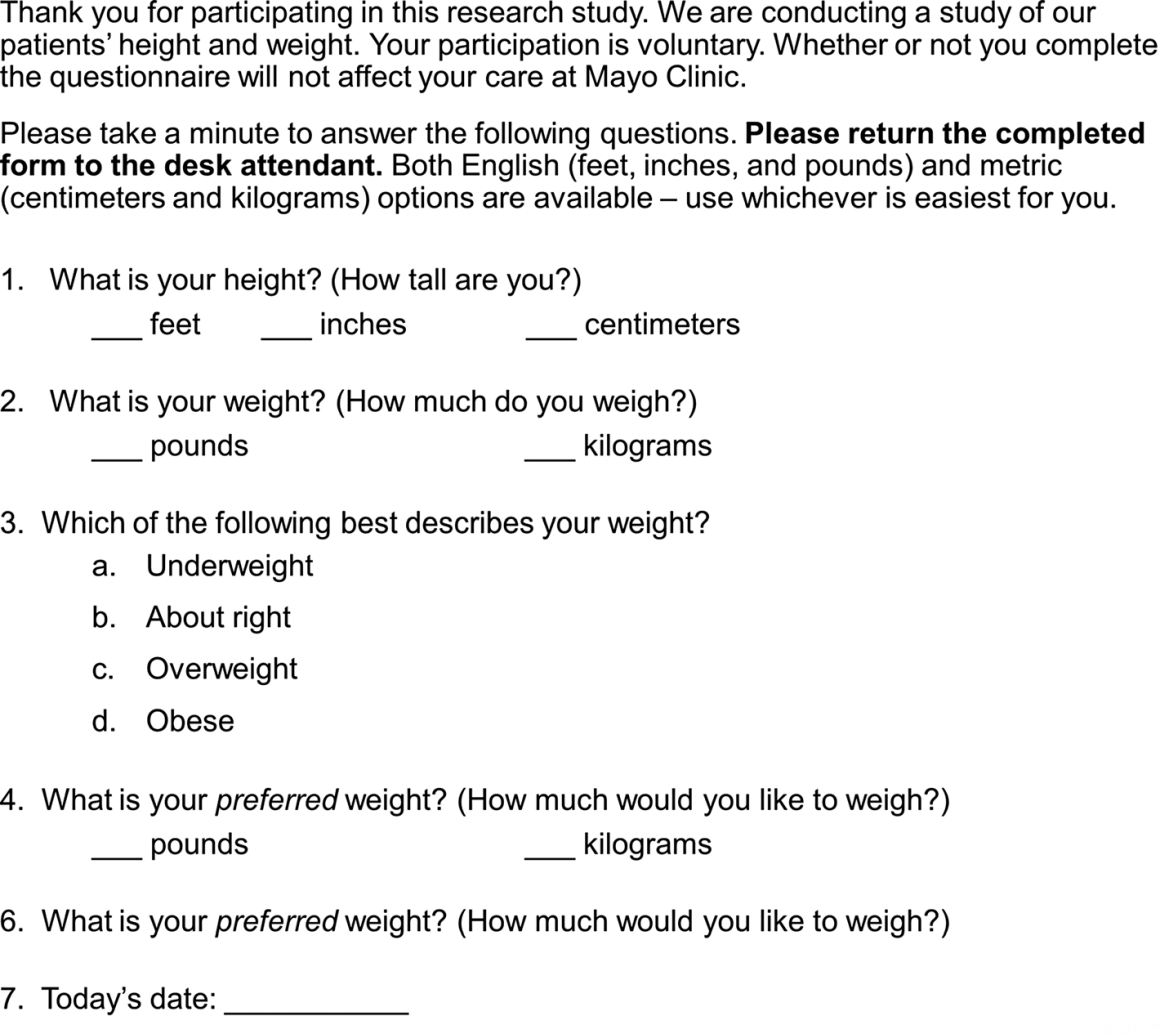
**Questionnaire administered to 508 consecutive patients seen in a general internal medicine clinic at Mayo Clinic in Rochester, Minnesota.**

The Mayo Clinic Institutional Review Board approved this cross-sectional study. Between November 9 and 20, 2009, consecutive adult outpatients who presented for appointments in the Division of General Internal Medicine at Mayo Clinic in Rochester, Minnesota, completed a 4-item questionnaire: 1) What is your height? 2) What is your weight? 3) Which of the following best describes your weight? (Possible answers were “underweight,” “about right,” “overweight,” “obese.”) 4) What is your preferred weight? (Figure 1). When applicable, self-reported heights and self-reported weights were converted to metric units.

After completing the questionnaire, the participants’ actual heights and weights were measured by trained clinical assistants using standard protocols. Height (cm) was measured using a precision electronic stadiometer (Seca, Hamburg, Germany). The participant stood straight and upright; shoes off and feet together; knees straight with back, buttocks and heels touching the back of the stadiometer; head in the Frankfurt plane; and arms hanging relaxed at sides of the body with palms facing thighs. The mobile horizontal part of the stadiometer touched the participant’s head with slight pressure. The participant was instructed to inspire and then the participant’s height was measured and recorded into his or her electronic medical record. Weight (kg) was measured using a precision electronic scale (Seca). The participant was instructed to remove his or her shoes and wear only light indoor clothing. The participant stood on the middle of the scale platform with weight equally distributed between the feet. The participant’s weight was then measured and recorded into his or her electronic medical record. Both devices were regularly calibrated.

BMIs were calculated using the following formula: BMI = (weight in kilograms)/([height in meters]^2^). Actual BMI was calculated using a participant’s actual height and actual weight. Self-reported BMI was calculated using a participant’s self-reported height and self-reported weight. Self-perceived BMI category was based on the third question of the questionnaire (“Which of the following best describes your weight?”). A participant’s desired BMI was calculated using the participant’s self-perceived height and desired weight. BMI categories established by the National Institutes of Health (NIH) were used: underweight (BMI < 18.5 kg/m2), normal (BMI 18.5-24.9 kg/m2), overweight (BMI 25–29.9 kg/m2), and obese (BMI > 30 kg/m2) [34].

Data from the participants’ electronic medical records were abstracted during December 2009 and January 2010. Participants’ medical records were also reviewed to determine the frequency of clinicians’ documented diagnoses of overweight and obesity in affected participants.

Descriptive statistics were used. Participants’ self-reported data were compared to their actual heights, actual weights, and actual BMI categories. The paired *t* test of continuous variables was used to compare average heights, weights, and BMIs between actual, self-reported, and desired categories. The Pearson χ^2^ test was used to compare differences in proportions in accuracy of self-perceived BMI categorization across actual BMI categories. The Cochrane-Armitage trend test was used to test for a linear trend in the proportion of patients who accurately self-perceived their BMI category according to actual BMI category. The Wald test of marginal homogeneity was used to compare proportions among actual, self-reported, self-perceived, and desired BMI categories. Analysis of variance (ANOVA) was used to test for a trend for the differences between actual and self-reported BMIs by increasingly higher BMI categories. ANOVA was also used to test for trends in average desired weight losses across actual and self-perceived BMI categories. Univariate logistic regression models were fit to assess associations between patient characteristics and accuracy of self-perceived BMI categorization. All variables were entered into a subsequent multivariate regression model. A *P* value of < .05 was considered significant. JMP 9 and SAS 9.2 (SAS Institute Inc, Cary, North Carolina) were used to carry out the statistical analyses.

## Results

The characteristics of the study participants are in Table 1. Overall, 508 patients (257 women, 251 men; mean age, 62.9 ± 14.9 years) participated in the study. A majority of patients lived in the United States (93%), had at least some college education (72%), and were never-smokers or former smokers (91%).

**Table 1:** **Characteristics of 508 consecutive patients seen in a general internal medicine clinic at Mayo Clinic in Rochester, Minnesota**

**Characteristic**	**No. (%)** ^**a**^
Age, mean ± SD, y	62.9 ± 14.9
Sex	
Female	257 (51)
Male	251 (49)
Home	
Minnesota	176 (35)
US, not Minnesota	296 (58)
International	35 (7)
Education level	
<High school graduate	31 (6)
High school graduate	100 (21)
Some college	125 (26)
College graduate	102 (21)
Postgraduate	122 (25)
Smoking status	
Never	244 (48)
Former	217 (43)
Current	44 (9)

^a^Values are number (percentage) of patients unless indicated otherwise.

Seventy percent of participants had overweight or obesity. The average self-reported height was significantly taller than the average actual height (170.2 ± 10.4 cm vs 169.4 ± 10.1 cm; *P* < .001). The average self-reported weight was significantly lower than the average actual weight (80.3 ± 20.1 kg vs 81.9 ± 21.1 kg; *P* < .001). Hence, the average self-reported BMI was significantly lower than the average actual BMI (27.6 ± 5.7 kg/m^2^ vs 28.3 ± 6.1 kg/m^2^; *P* < .001). The average desired weight (72.5 ± 14.8 kg) was significantly lower than the average self-reported weight and the average actual weight (*P* < .001 for both comparisons). The average desired BMI was significantly lower than the average actual and self-reported BMI (*P* < .001 for both comparisons). The overall average desired weight loss was 9.4 ± 12.3 kg, which, if achieved, would result in an average desired BMI of 24.8 ± 3.2 kg/m^2^. Similar results were obtained when the data were analyzed by sex (data not shown).

Distribution of the patients by their actual, self-reported, self-perceived, and desired BMI categories is in Table 2. Thirty-two percent of the patients actually had obesity. However, only 6% of patients perceived they were obese. The proportions of patients categorized as underweight, normal weight, overweight, and obese by self-reported BMI, self-perceived BMI category, and desired BMI differed significantly compared with the proportions of patients categorized as underweight, normal weight, overweight, and obese by actual BMIs (*P* < .001 for all 3 comparisons). Similarly, for normal-weight, overweight, and obese patients, self-reported BMIs were significantly less than actual BMIs (*P* < .001 for each comparison). In addition, these differences increased significantly the higher the actual BMI category (*P* < .001 for trend). Similar results were obtained when the data were stratified by sex (Table 3).

**Table 2:** **Distribution of 508 patients by actual, self-reported, self-perceived, and desired body types** ^**a**^

**NIH BMI category**	**Actual**	**Self-reported**	**Self-perceived**	**Desired**
Underweight	11 (2)	12 (2)	33 (6)	5 (1)
Normal	138 (27)	160 (31)	189 (37)	255 (50)
Overweight	193 (38)	197 (39)	248 (49)	194 (38)
Obese	165 (32)	132 (26)	33 (6)	26 (5)
No data	1 (0)	5 (1)	4 (1)	28 (6)

*Abbreviations: BMI* body mass index, *NIH* National Institutes of Health.

^a^Values are number (percentage) of patients. *P* < .001 for all 3 comparisons of self-reported, self-perceived, and desired body type distributions vs actual body type distribution (Wald test of marginal homogeneity).

**Table 3:** **Actual BMI vs self-reported BMI by actual body type**

**NIH BMI category**	**Actual BMI, kg/m** ^**2a**^	**Self-reported BMI, kg/m** ^**2a**^	**Difference, kg/m** ^**2b**^	***P*** **value** ^**c**^
All patients (N = 508)				
Underweight	17.3 ± 0.7	17.4 ± 0.8	−0.1	.70
Normal	22.5 ± 1.6	22.2 ± 1.6	0.3	<.001
Overweight	27.4 ± 1.4	26.7 ± 1.6	0.7	<.001
Obese	35.1 ± 5.1	33.8 ± 4.9	1.3^d^	<.001
Women (n = 257)				
Underweight	17.3 ± 0.7	17.4 ± 0.8	−0.1	.70
Normal	22.3 ± 1.6	22.0 ± 1.8	0.3	.001
Overweight	27.2 ± 1.4	26.5 ± 1.6	0.7	<.001
Obese	35.2 ± 4.4	34.1 ± 4.1	1.1^d^	<.001
Men (n = 251)				
Underweight	NA	NA	NA	NA
Normal	23.0 ± 1.5	22.7 ± 1.3	0.3	.002
Overweight	27.6 ± 1.4	26.8 ± 1.5	0.7	<.001
Obese	35.0 ± 5.6	33.5 ± 5.4	1.5^d^	<.001

*Abbreviations: BMI* body mass index, *NA* not applicable, *NIH* National Institutes of Health.

^a^Values shown are mean ± SD.

^b^Analysis of variance for trend of differences.

^c^Matched pairs *t* test comparing actual BMI vs self-reported BMI by actual body type.

^d^ *P* < .001 using analysis of variance for trend of differences.

**Figure 2:**
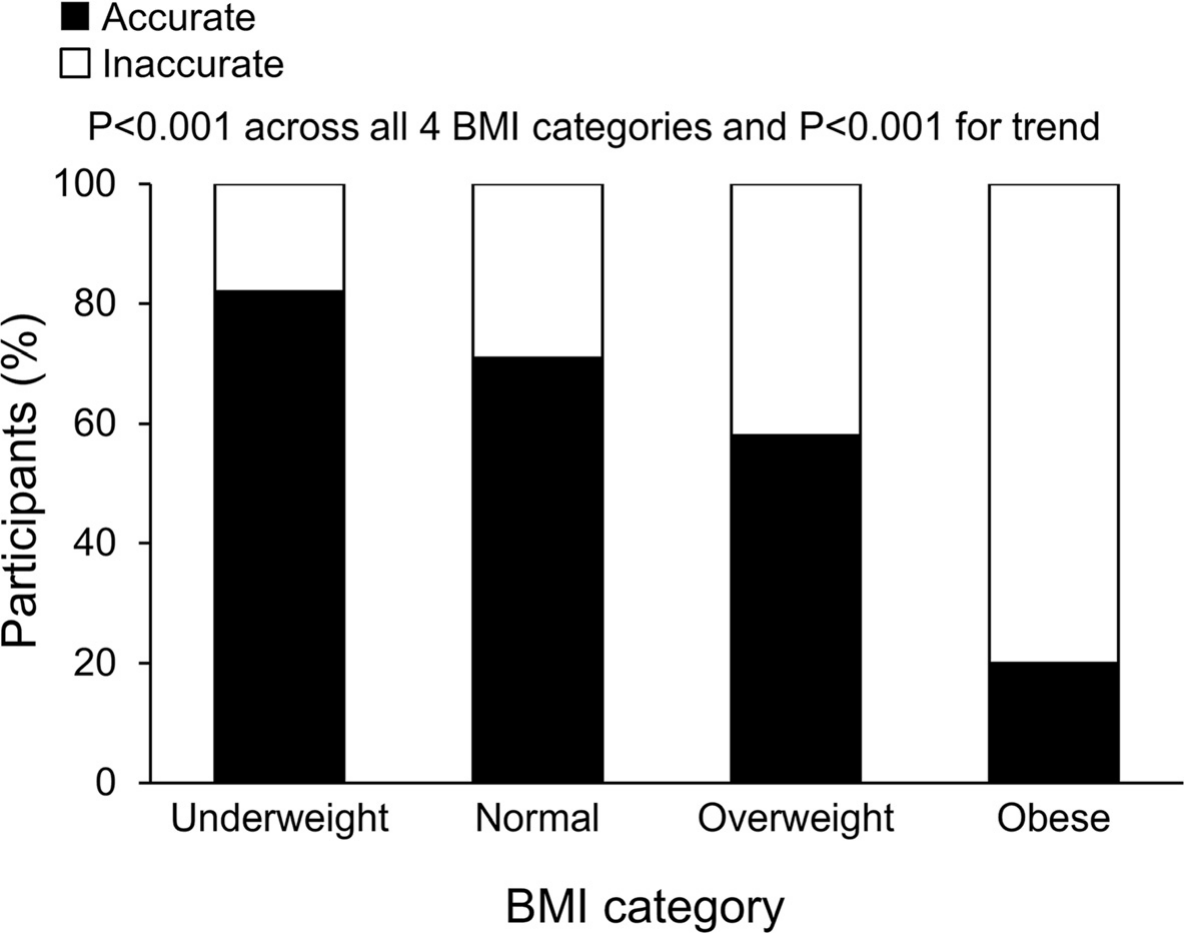
**Accuracy of self-perceived BMI category decreased by actual BMI category among 508 consecutive patients seen in a general internal medicine clinic at Mayo Clinic in Rochester, Minnesota.**

Accuracy of self-perceived BMI category decreased with higher actual BMIs (Figure 2 and Table 4). For example, 98 (71%) of 138 normal-weight participants accurately perceived their BMI category compared with 112 (58%) of 194 overweight participants and 32 (20%) of 164 obese participants (*P* < .001 across all 4 BMI categories and *P* < .001 for trend). Of the 82 inaccurate overweight participants, 80 (98%) perceived they were normal weight and 2 perceived they were underweight. Of the 132 inaccurate obese participants, 119 (90%) perceived they were overweight and 9 (7%) perceived they were normal weight (for 4 participants, no information was available). Similar results were obtained when the data were stratified by sex.

**Table 4:** **Accuracy of self-perceived BMI compared with actual BMI category among 508 consecutive patients** ^**a**^

	**Actual BMI category**				
**Accuracy of self-perceived BMI**	**Underweight**	**Normal**	**Overweight**	**Obese**	***P*** **value** ^**b**^
All patients (n = 508)					<.001
Accurate	9 (82)	98 (71)	112 (58)	32 (19)	
Inaccurate	2 (18)	40 (29)	82 (42)	132 (81)	
Women (n = 257)					<.001
Accurate	9 (82)	65 (69)	62 (76)	17 (24)	
Inaccurate	2 (18)	29 (31)	19 (24)	54 (76)	
Men (n = 251)^c^					<.001
Accurate	NA	33 (75)	50 (44)	15 (16)	
Inaccurate	NA	11 (25)	63 (56)	78 (84)	

*Abbreviations: BMI* body mass index, *NA* not applicable.

^a^Across all 4 categories and for trend.

^b^Values are percentages of patients.

^c^Data were missing for 1 patient.

Univariate and multivariate analyses were done to determine factors associated with increased accuracy of self-perceived BMI category (Table 5). Univariate analyses revealed that younger age, female sex, higher education level, and lower actual BMI were significantly associated with increased accuracy of self-perceived BMI category. Multivariate analysis revealed that female sex, higher education level, smoking status (trends for quitters more accurate than never-smokers and never-smokers more accurate than smokers), and lower actual BMI were significantly associated with increased accuracy of self-perceived BMI category. Notably, interaction between actual BMI and sex for accuracy of perceived BMI category was not found (*P* = .78).

**Table 5:** **Univariate and multivariate associations between patient characteristics and accuracy of self-perceived BMI type**

	**Univariate**		**Multivariate**	
**Patient characteristic**	**OR (95% CI)**	***P*** **value**	**OR (95% CI)**	***P*** **value**
Age, per year	0.99 (0.97-1.00)	.02	0.99 (0.98-1.01)	.22
Male sex	0.43 (0.31-0.62)	<.001	0.52 (0.34-0.77)	.001
Residence		.94		.70
Non-MN vs MN	1.06 (0.51-2.20)	.88	1.08 (0.47-2.55)	.85
International vs MN	0.95 (0.66-1.39)	.80	0.86 (0.57-1.30)	.48
Education		.003		.03
HS graduate vs < HS graduate	1.29 (0.56-3.12)	.56	1.34 (0.55-3.43)	.52
Some college vs < HS graduate	2.07 (0.92-4.91)	.08	1.96 (0.82-4.92)	.13
College graduate vs < HS graduate	3.39 (1.48-8.24)	.004	2.96 (1.22-7.56)	.02
Post-graduate vs < HS graduate	2.39 (1.06-5.71)	.04	2.45 (1.03-6.12)	.04
Smoking status		.44		.04
Former vs never smoker	1.18 (0.82-1.71)	.37	1.49 (0.99-2.27)	.06
Smoker vs never smoker	0.81 (0.42-1.54)	.53	0.66 (0.31-1.37)	.27
Actual BMI, per single BMI unit	0.92 (0.88-0.95)	<.001	0.93 (0.89-0.96)	<.001

*Abbreviations: BMI* body mass index, *CI* confidence interval, *HS* high school, *MN* Minnesota, *OR* odds ratio.

**Figure 3:**
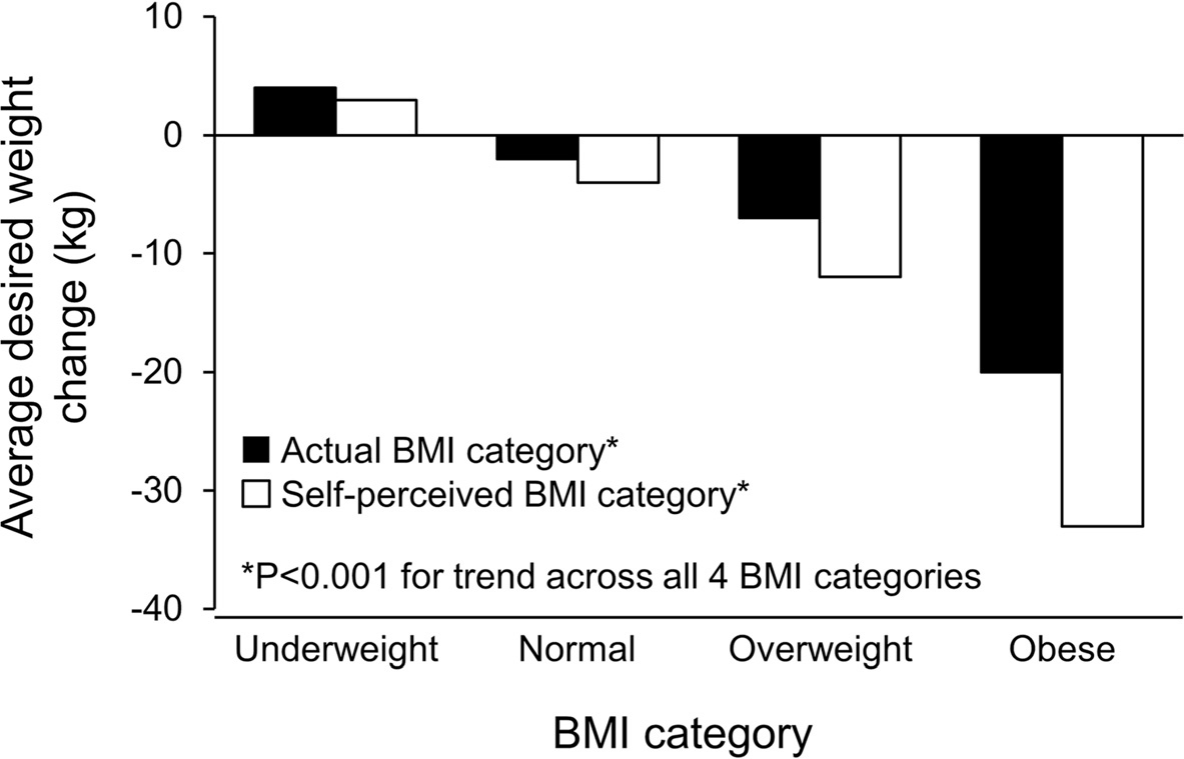
**Average desired weight loss by actual BMI and self-perceived BMI category among 508 consecutive patients seen in a general internal medicine clinic at Mayo Clinic in Rochester, Minnesota.**

Desired weight loss increased with higher actual BMI and self-perceived BMI category (Figure 3). For example, participants who actually were normal weight, overweight, and obese desired weight losses of 1.7 ± 4.2 kg, 7.1 ± 8.4 kg, and 19.7 ± 13.9 kg, respectively (*P* < .001 for trend). Participants who self-perceived they were normal weight, overweight, and obese desired weight losses of 3.8 ± 8.5 kg, 12.2 ± 8.0 kg, and 33.8 ± 21.2 kg, respectively (*P* < .001 for trend). Participants who actually were underweight and those who self-perceived they were underweight desired weight gain. Similar results were obtained when the data were analyzed by sex (data not shown).

Of the 165 patients who actually were obese, only 40 (24%) had obesity documented as a diagnosis in their medical records by their clinicians. Of the 133 patients who were obese based on their self-reported heights and weights, only 37 (28%) had obesity documented. However, of the 33 patients who perceived themselves as obese, 14 (42%) had obesity documented.

## Discussion

To our knowledge, this is the first study to compare adult ambulatory outpatients’ self-reported heights, weights, and BMIs, desired weights and BMIs, and self-perceived BMI categories with their actual heights, weights, and BMIs and determine factors associated with patient accuracy of self-perceived BMI categorization and rates of clinicians’ documented diagnoses of obesity. The key findings were that 1) 70% of the patients had overweight or obesity; 2) the overall average self-reported height and weight were significantly taller and lighter than the average actual height and weight, resulting in an average self-reported BMI that was significantly less than the average actual BMI; 3) the average desired weight loss was substantial and, if achieved, would result in a normal average BMI; 4) many overweight and obese patients inaccurately perceived their BMI category, and accuracy decreased significantly with higher actual BMI category; 5) desired weight loss increased significantly with higher self-perceived and actual BMI categories; 6) female sex, higher education level, smoking status, and lower actual BMI were associated with increased accuracy of self-perceived BMI categorization; and 7) only one-quarter of obese patients had obesity as a diagnosis documented by their clinicians in their medical records.

The finding that 70% of the patients in our study had overweight or obesity reflects the epidemic of unhealthy weight in the United States [10,31]. A number of studies have assessed patient self-reported height [6-9,15-17,20,21,23-27,35,36] self-reported weight [6-9,15-17,20,21,23-27,35,36] and self-reported BMI (calculated on the basis of self-reported height and weight) [6-9,15-17,20,21,23-27,35,36]. However, only 4 studies have compared self-reported height, weight, and BMI with actual height, weight, and BMI [7,16,25,36]. Consistent with the results of prior studies [24-26,35], the patients in our study reported being taller and lighter than they actually were. Although self-reported heights and weights may be useful for epidemiologic studies, our results suggest that clinicians should not rely on these self-reported data for clinical decision making. Instead, actual measurements of heights and weights should be used. Notably, the US Preventive Services Task Force (USPSTF) recommends screening all adults for obesity by calculating BMI from measured weight and height [37]. (The USPSTF uses the same BMI categories as NIH, as described in the Methods and Procedures section [34]).

More concerning is the finding that many overweight and obese participants in our study inaccurately self-perceived their BMI categories; nearly all inaccurate participants perceived they were in a lower BMI category than they actually were. Furthermore, the higher the actual BMI category, the more inaccurate was the self-perceived BMI category, suggestive of a dose–response relationship. Higher education, smoking status, and female sex were associated with greater accuracy of self-perceived BMI category. According to the Health Belief Model, an overweight or obese person must recognize his or her unhealthy weight and its associated health risks before he or she will modify unhealthy lifestyle behaviors such as lack of exercise and poor diet (eg, processed foods, fats, decreased fruits and vegetables) and lose weight [28].

Nonetheless, most of the participants in our study desired to lose weight, and the average desired weight loss was substantial (9.4 kg). If the participants achieved this weight loss, the overall average BMI would be normal (24.8 kg/m^2^). This novel finding suggests that, despite inaccurate self-reporting of heights and weights and self-perceived BMI categories, patients recognize the need for healthy weight. Unsurprisingly, desired weight loss was associated with self-perceived BMI category. For example, patients who perceived they were overweight or obese desired more weight loss than patients who actually were overweight or obese. Health care professionals can leverage these findings in order to discern patients’ self-perceived weights, correct misperceptions, and make recommendations regarding management and achieving healthy weight. Overweight and obese patients who are counseled about their unhealthy weight by their health professionals may have more accurate self-perceptions of weight and may be more likely to attempt to lose weight [7,29,30]. Furthermore, evidence suggests that behavioral interventions, with or without pharmacologic interventions, result in substantial weight loss [37,38]. Although such interventions may not result in a given patient’s desired weight loss, it is important to recognize that modest weight loss (5% to 10%) can mitigate cardiovascular risk factors [39]. However, for these interventions to be effective, clinician diagnosis and patient self-perception of unhealthy weight and its health risks are essential.

Although health care professionals are uniquely positioned to help overweight and obese patients recognize their unhealthy weight, these professionals, like those in our study, often fail to do so. Only one-quarter of the participants in our study who actually were obese had obesity documented as a diagnosis in their medical records by their clinicians. This phenomenon of obesity “hiding in plain sight of the physician” has been observed previously at our institution [29] and elsewhere [31-33]. If overweight and obese patients do not perceive their weight as unhealthy and overweight and obesity hide in plain sight, then it is unlikely unhealthy weight will be addressed by health care professionals before weight-related health events (eg, diabetes, myocardial infarction, sleep apnea, osteoarthritis) occur.

Why do health care professionals fail to recognize and diagnose overweight and obesity? As mentioned, some clinicians do not regard obesity as a disease [5]. Yet, now the AMA does [4]. Furthermore, until recently, Medicare did not reimburse for obesity counseling. Thus, there was no financial incentive for diagnosing obesity. Now, such an incentive exists [40]. Some clinicians may regard counseling overweight and obese patients to engage in healthy behaviors and lose weight as futile. However, evidence suggests that behavioral interventions (with or without pharmacologic interventions) are effective [37]. Barriers cited by health care professionals at our institution include lack of time to discuss patients’ weights, other clinical priorities, perceived lack of effective treatments, provider unpreparedness to discuss obesity, patient sensitivity to the term “obesity”, and other factors (Cook KE, Salerno MS, Williams BJ, Klauer KM, Hensrud DD, Collazo-Clavell ML, Hurt RT, Wermers RA, Kebede EB, Mueller PS, unpublished data). Other barriers include lack of infrastructure to meet overweight and obese patients’ needs, patients’ concerns about stigma, and “antifat bias” by clinicians [33].

Health care institutions should implement measures that address these barriers (eg, electronic medical record prompts that trigger clinicians to inform overweight and obese patients of their unhealthy weights and the associated risks and discuss treatment options with affected patients). Clinicians uncomfortable with counseling affected patients about overweight and obesity should be offered communication training. Institutions should also provide resources that assist clinicians in providing the multimodal, high-intensity counseling and follow-up that are needed to help patients lose weight.

This study has a number of limitations. First, the average age of the participants was 63 years, more than a third lived in Minnesota, and most had at least a high school education. Second, we were unable to determine patient ethnic and socioeconomic status; these factors have been associated with varied self-perception of weight and self-recognition of obesity [6,9,16,21-23]. Hence, the same study conducted with a patient population with different characteristics might yield different results. Future studies should include populations with broader demographics to allow for analysis between ethnic groups, levels of education and socioeconomic status, and other patient characteristics. Third, some people with BMIs higher than 25 kg/m^2^ have low body fat. Also, body fat distribution affects risk for comorbid disease. In overweight and obese patients, higher waist circumference, an indicator for central obesity, is associated with higher risk [34]. Our study would have been strengthened by including waist circumference, skinfold measurement, and body fat composition analysis as variables.

## Conclusions

Ambulatory patients inaccurately report their heights and weights and inaccurately perceived their BMI categories. Health care professionals should not rely on patient self-reported height and weight data and, instead, should measure patients’ heights and weights. Professionals should inform patients of their BMI categories and advise overweight and obese patients of the health consequences of their unhealthy weights (and prescribe treatment plans as appropriate). Health care institutions should employ measures that increase clinician recognition and diagnosis of overweight and obesity.

## Abbreviations

AMA: American Medical Association
ANOVA: Analysis of variance
BMI: Body mass index
NIH: National Institutes of Health
USPSTF: US Preventive Services Task Force
